# The loss of health status in rheumatoid arthritis and the effect of biologic therapy: a longitudinal observational study

**DOI:** 10.1186/ar2944

**Published:** 2010-03-02

**Authors:** Frederick Wolfe, Kaleb Michaud

**Affiliations:** 1 National Data Bank for Rheumatic Diseases, 1035 N. Emporia, Suite 288, Wichita, KS 67214, USA

## Abstract

**Introduction:**

The long-term course of rheumatoid arthritis (RA) in terms of health status is not well understood, nor is the degree of effectiveness of biologic therapy in the community. We modeled the progression of loss of health status, and measured incremental costs and effectiveness of biologic therapy in the community.

**Methods:**

We studied change in function and health status in 18,485 RA patients (135,731 observations) at six-month intervals for up to 11 years, including a group of 4,911 patients (59,630 observations) who switched to biologic therapy from non-biologic therapy. We measured the SF-36 Physical Component (PCS) and Mental Component (MCS) Summary scales, the EQ-5D health utility scale, and the Health Assessment Questionnaire (HAQ) disability scale; and we calculated treatment and direct medical costs.

**Results:**

RA onset caused an immediate and substantial reduction in physical but not mental health status. Thereafter, the progression of dysfunction in RA was very slow (HAQ 0.016 units and PCS -0.125 units annually), only slightly worse than the age and sex-adjusted US population. We estimated biologic treatment to improve HAQ by 0.29 units, PCS by 5.3 units, and EQ-5D by 0.05 units over a 10-year period. The estimated incremental 10-year total direct medical cost for this benefit was $159,140.

**Conclusions:**

Biologic therapy retards RA progression, but its effect is far less than is seen in clinical trials. In the community, cost-effectiveness is substantially less than that estimated from clinical trial data. The study results represent the incremental benefit of adding biologic therapy to optimum non-biologic therapy.

## Introduction

Biologic therapy for rheumatoid arthritis (RA) has been shown to be efficacious in multiple clinical trials [1-10]. This efficacy extends from composite measures that include physician, patient, and laboratory tests such as the Disease Activity Index-28 (DAS28) [11] and the American College of Rheumatology (ACR) improvement criteria [12], to imaging studies [1], as well as to purely patient-based assessments such as the Health Assessment Questionnaire disability index (HAQ) and the Short Form-36 (SF-36) [13]. Efficacy data, from these trials, usually based on the HAQ or health utility scales [14], are used in cost-effectiveness studies and assessments of costs per Quality Adjusted Life-Years (QALYs) [15], and extrapolated to future but unobserved results.

The degree of effectiveness of biologic therapy treatments in clinical practice in the community, however, has not been established, but effectiveness studies often show less benefit than efficacy studies. The idea of effectiveness (Does it work in the community?) is somewhat different from the idea of efficacy (Does it work in the clinical trial setting?). In addition, effectiveness implies sustained improvement in generally unselected populations (Does it *really *work?), and effectiveness studies are concerned with the degree of improvement and, sometimes, with the cost of improvement, areas that we investigate in the current study.

With respect to RA treatment, there is another important difference between community effectiveness studies and randomized clinical trials. In the community, biologic therapy is added to generally effective therapy already being used. Thus, observed benefit from biologic therapy in the community represents the incremental benefit of adding biologic therapy. Observational studies do not represent an alternative to the experimentation of randomized clinical trials, but rather represent a set of complementary approaches, as the external validity, or *generalizability*, of the results of randomized trials is often low [16,17].

Health status and functional status are central components of RA outcomes. They are meaningful to patients and form the basis of cost-effectiveness pronouncements [14]. In addition, because it is almost impossible to carry out large, long-term, population-based studies that include imaging and reliable physician assessments, effectiveness studies in RA are more easily executed using patient assessments that measure health-related quality of life and function.

As background to the question of biological therapy effectiveness in RA, we first describe the lifetime course of health status in RA and then the 10-year observed course of health status in RA patients in order to provide information about RA and to provide further validation for the study methods. We then examine the incremental benefit of biologic therapy in an unselected population of patients with RA by following patients longitudinally who switch from non-biologic treatment of at least six months duration to biologic treatment during their ordinary clinical care. Thus, patients provide their own controls. We assess patients continuously in both periods using semiannual mailed and web-based questionnaires, using PCS, MCS, HAQ, and EQ-5D as the study outcome measures. We calculate the rates of progression of loss of health status in both treatment periods, and we compare the rates to determine treatment effect, adjusting for important socio-economic differences; we also determine direct treatment and total medical costs. Essentially, the question we ask is, 'What is the effect of biologic therapy on the functional and health status of patients starting this therapy compared with their previous course?'

## Materials and methods

We studied 18,485 adult patients with RA who participated in the National Data Bank for Rheumatic Diseases (NDB) longitudinal study of RA outcomes. Participants are volunteers, recruited from the practices of US rheumatologists, who complete mailed or Internet questionnaires about their health at six-month intervals. They are not compensated for their participation. The diagnosis of RA is made by the patient's rheumatologist. Patients who were recruited to participate in the NDB as they started a biologic therapy, specifically as part of a biologic safety registry, were excluded from this study because of the possibility of severity bias. The NDB utilizes an open cohort design in which patients are enrolled continuously.

**Figure 1:**
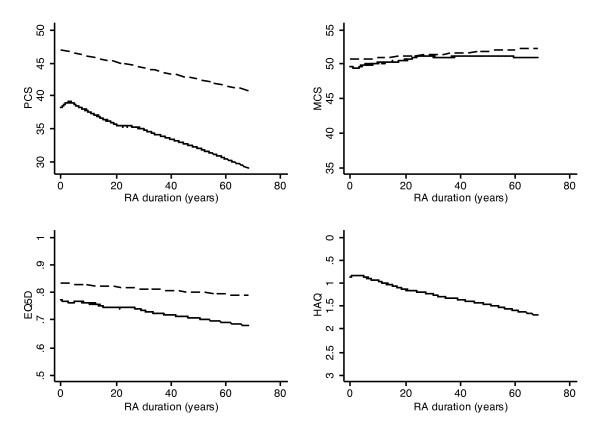
**The change in health status over the lifetime course of RA**. The change in health status over the lifetime course of 18,485 RA patients for PCS (upper left), MCS (upper right), EQ-5D (lower left), and HAQ (lower right). The dashed lines for PCS, MCS, and EQ-5D represent age and sex adjusted population normative data. See methods for details. HAQ, Health Assessment Questionnaire; PCS, SF-36 Physical Component Summary; MCS, SF-36 Mental Component Summary.

Patients were assessed on a semiannual basis between 1998 and 2009. At each assessment we obtained treatment and demographic data by patient self-report. Patients were considered to be on biologic therapy if they used any of the following treatments during the time of the study: etanercept, infliximab, adalimumab, abatacept, certolizumab pegol, or rituximab. For the study health status and function measures, we calculated the physical (PCS) and mental (MCS) component summary scores from the SF-36 version 1 according to the authors' recommendations [18,19]. The primary time period of the SF-36 questionnaire was four weeks. We used the EQ-5D to determine health utilities. The EQ-5D is a five-item questionnaire that assesses function (three questions), mood (one question) and pain (one question) [20]. Scoring was accomplished using US tariffs (weights) [21,22]. US and European scores are not interchangeable, with US scores being approximately 0.11 units greater [23]. To measure functional status, we used the Health Assessment Questionnaire disability index (HAQ) [24]. The HAQ has 34 questions, including 20 activities of daily living items and 14 aids and devices. The SF-36, EQ-5D, and the HAQ have been used extensively in RA research. To compare study patients with age and sex matched patients in the US population (Figure 1) we used published normative data for the PCS and MCS [25] and EQ-5D [21].

We computed a comorbidity score based on the presence of pulmonary disorders, myocardial infarction, other cardiovascular disorders, stroke, hypertension, diabetes, spine/hip/leg fracture, depression, GI ulcer, other GI disorders, and cancer, as previously described [26].

Direct medical costs, adjusted to 2007, were determined from semi-annual hospitalization, treatment and utilization data, and applied using US Centers for Medicare and Medicaid Services fee schedules for procedures, and average wholesale prices for treatments, for the corresponding year, as previously reported [27].

### Statistical methods

**Figure 2:**
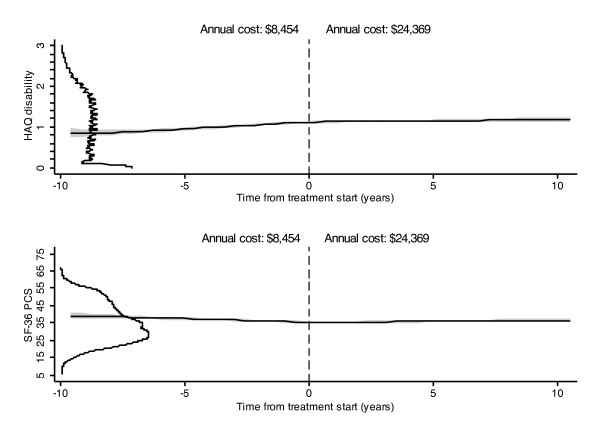
**Changes in HAQ and PCS in patients treated with biologic therapy**. Changes in HAQ and PCS in patients who started a biologic therapy at time 0. Horizontal graphs at y-axis represent curves of the distribution of values for HAQ (above) and PCS (below) at time 0. *Annual cost *is the annual cost calculated for the 10-year duration before and the 10-year duration after the start of biologic therapy. HAQ, Health Assessment Questionnaire; PCS, SF-36 Physical Component Summary; MCS, SF-36 Mental Component Summary.

As the course of RA may extend to more than 60 years, no study can encompass the duration of the illness, and many of the study instruments we used have been in common use for less than 25 years [28,29]. To partially overcome this problem in describing the course of RA (Figure 1 and Table 1), we used an accelerated cohort design [30]. In this design the time metric is the duration of RA, not the semi-annual wave of study assessments. A potential problem with accelerated cohort design studies is data sparseness. However, there were sufficient patients in this study to avoid this problem: 605 patients had RA for ≥40 years and 128 had it for ≥50 years. In long duration longitudinal studies using this design, right censoring may be a problem in that the patients with the longest RA duration may be *survivors *and differ systematically from non-participants (non-survivors). We used the accelerated cohort design in order to be able to describe long duration RA health status. Readers may choose to examine graphic data at maximum durations of 30 or 40 years to avoid problems that might be caused by this censoring. In addition, 1,514 patients had RA for ≤2 years and 4,174 for ≤4 years when first enrolled in the NDB. The major part of the study (Tables 2 and 3, Figure 2), which deals with observed time rather than duration of RA, utilized semi-annual cohort assessments over a maximum of 11 years.

**Table 1:** Annualized lifetime rates of progression and levels of disability and health status in rheumatoid arthritis

Variable	All patients (N = 18,485) Rate of progression Rate (95% CI)	Values at RA duration Of 0 years (onset)* Mean (95% CI)	Values at RA duration of 10 years* Mean (95% CI)	Values at RA duration of 20 years Mean (95% CI)
HAQ	0.016 (0.015, 0.017)	0.84 (0.83, 0.86)	1.00 (0.99, 1.01)	1.16 (1.15, 1.18)
PCS	-0.125 (-0.142, -0.108)	37.9 (37.6, 38.1)	36.6 (36.5, 36.8)	35.4 (35.2, 35.6)
MCS	0.047 (0.031, 0.063)	48.5 (48.3, 48.8)	49.0 (48.8, 49.1)	49.4 (49.4, 49.6)
EQ-5D	-0.001 (-0.001, -0.000)	0.748 (0.742, 0.754)	0.739 (0.736, 0.743)	0.731 (0.727, 0.735)

Positive rates indicate worsening HAQ disability. Negative rates indicate worsening health status for MCS, PCS, and EQ-5D. Values at 0, 10, and 20 years RA duration are estimated from the mixed model regression analysis.

HAQ, Health Assessment Questionnaire; PCS, SF-36 Physical Component Summary; MCS, SF-36 Mental Component Summary.

**Table 2:** Annualized *observed rates *of progression of disability and loss of health status in rheumatoid arthritis

Variable	All patients (N = 18,485) Rate (95% CI)	Biologics never used (N = 10,265) Rate (95% CI)	Biologics ever used (N = 8,220) Rate (95% CI)
HAQ	0.013 (0.010, 0.015)	0.016 (0.013, 0.019)	0.010 (0.007, 0.013)
PCS	0.035 (0.001, 0.069)	-0.003 (-0.048, 0.041)	0.068 (0.022, 0.114)
MCS	0.039 (0.008, 0.069)	0.001 (-0.041, 0.063)	0.044 (-0.003, 0.090)
EQ-5D	0.001 (0.001, 0.002)	-0.000 (-0.001, 0.001)	0.002 (0.012, 0.003)

Annualized *observed rates *of progression of disability and loss of health status in 18,485 RA patients during up to 10 years of observation.

Positive rates indicate worsening HAQ disability. Negative rates indicate worsening health status for MCS, PCS, and EQ-5D.

HAQ, Health Assessment Questionnaire; PCS, SF-36 Physical Component Summary; MCS, SF-36 Mental Component Summary.

**Table 3:** Effect of biologic therapy on rheumatoid arthritis progression

Variable	N	Pre biologic therapy Rate (95% CI)	Post biologic therapy Rate (95% CI)	Rate difference Rate (95% CI)
HAQ (all)	4,911	0.032 (0.027, 0.036)	0.003 (0.000, 0.006)	-0.029 (-0.023, -0.034)
HAQ (on)	3,829	0.031 (0.026, 0.036)	-0.001 (-0.004, 0.002)	-0.033 (-0.026, -0.039)
HAQ (DC)	1,082	0.033 (0.021, 0.044)	0.013 (0.008, 0.017)	0.020 (0.007, 0.033)
				
PCS (all)	4,911	-0.353 (-0.432, -0.273)	0.179 (0.129, 0.229)	-0.532 (-0.634, -0.430)
PCS (on)	3,829	-0.348 (-0.436, -0.260)	0.261 (0.198, 0.323)	-0.608 (-0.725, -0.491)
PCS (DC)	1,082	-0.396 (-0.591, -0.201)	-0.003 (-0.082, 0.075)	-0.393 (-0.619, -0.166)
MCS (all)	4,911	-0.096 (-0.176, -0.016)	0.000 (-0.056, 0.057)	-0.096 (-0.197, 0.005)
				
MCS (on)	3,829	-0.124 (-0.211, -0.037)	0.052 (-0.012, 0.116)	-0.176 (-0.289, -0.063)
MCS (DC)	1,082	0.015 (-0.083, 0.212)	-0.117 (-0.226, -0.008)	0.132 (-0.104, 0.368)
				
EQ-5D (all)	3,997	-0.003 (-0.005, -0.000)	0.002 (0.001, 0.003)	-0.005 (-0.008, -0.002)
EQ-5D (on)	3,031	-0.003 (-0.006, 0.000)	0.003 (0.002, 0.004)	-0.006 (-0.009, -0.002)
EQ-5D (DC)	966	-0.001 (-0.007, 0.004)	-0.001 (-0.003, 0.002)	-0.001 (-0.007, 0.006)
				
**All patients**		**Pre biologic therapy** **Mean (95% CI)**	**Post biologic therapy** **Mean (95% CI)**	**Cost difference** **Mean (95% CI)**

Total Costs	4,911	$8,454 (8,140, 8,769)	$24,369 (21,172, 24,565)	$15,914 (15,543, 16,285)
Drug Costs	4,911	$4,681 (4,401, 4,961)	$20,401 (20,226, 20,576)	$15,720 (15,390, 16,051)
				
**Variable**	**N**	**Estimated scores** **Time = -10 years** **Mean (95% CI)**	**Actual scores** **Time = 0 years** **Mean (95% CI)**	**Estimated scores** **Time = +10 years** **Mean (95% CI)**

HAQ	4,911	1.02 (0.99, 1.05)	1.13 (1.11, 1.15)	1.24 (1.21, 1.27)
PCS	4,911	35.2 (34.7, 35.6)	35.4 (35.1, 25.7)	35.7 (35.2, 36.1)
MCS	4,911	50.2 (49.8, 50.7)	49.8 (49.5, 50.1)	49.3 (48.9, 49.8)
EQ-5D	3,997	0.721 (0.709, 0.733)	0.730 (0.724, 0.736)	0.740 (0.730, 0.749)

Effect of biologic therapy on annual rates of progression of disability, loss of health status, and costs in patients prior to and after the start of biologic therapy in RA.

All, all patients; on, on biologics at study close; DC, discontinued biologic before study close.

Positive rates indicate worsening HAQ disability. Negative rates indicate worsening health status for MCS, PCS, and EQ-5D. A negative rate difference indicates improvement. Times are relative to biologic therapy initiation.

HAQ, Health Assessment Questionnaire; PCS, SF-36 Physical Component Summary; MCS, SF-36 Mental Component Summary.

#### Missing data

Missing data occurred through two mechanisms in this study: 1) when patients did not complete or validly complete a questionnaire item (mechanism 1) and 2) when the item was not part of the assessment questionnaire (mechanism 2). The NDB made use of three types of questionnaires. All participants completed at least once a comprehensive 28-page questionnaire that included all study questions. Over the course of the study the comprehensive questionnaire was completed at 92.7% of observations, and had missing data rates for HAQ, PCS, and MCS of 0.4%, 3.2%, and 3.2%, respectively (mechanism 1). A short questionnaire and an even shorter (brief) questionnaire were completed by 6.5% and 1.0% of patients. These questionnaires did not include the PCS, MCS, HAQ, EQ-5D, or income questions (mechanism 2). Patients completing shorter questions are older and have lower health status compared with patients completing the comprehensive questionnaire. Considering all questionnaires, the *overall *missing data rate for HAQ, PCS, and MCS was 8.1%, 10.8%, and 10.8%, respectively. The missing data rate for total household income (comprehensive questionnaire) was 4.2% (mechanism 1). Because of the possibility that excluding data from short and brief questions would introduce unacceptable bias, we elected to impute the missing variables. To replace missing values, we used multiple imputation by chained equations (ICE) to create five multiple imputed datasets for analyses [31], and we combined data according to Rubin's rules [32]. The EQ-5D was not collected by the NDB until mid 2002, and 45.9% of EQ-5D values were missing for that reason. We elected to not impute missing data for the EQ-5D.

#### Specific analyses

In the main analyses we used a two-level mixed model with the individual patient as the second level and, depending on the analysis, different duration variables as the random intercept and random slope. In the RA duration model (Table 1 and Figure 1), the fixed covariates were age at RA onset and sex. In the observed duration model (time in the NDB) (Tables 2 and 3, Figure 2), the fixed effect covariates included: comorbidity, sex, year of RA onset, age at first NDB participation, education level, sex, and total household income. In the treatment model, duration was calculated as time before and time after treatment, with a range. In this model the fixed covariates were the same as in the observed duration model.

In all of these analyses, we first explored a series of different functional forms for time (duration of RA, duration in study, duration on treatment) and determined that non-linear terms offered no advantage over linear terms. In addition, we conducted sensitivity analyses using fully imputed data, singly imputed data, and case-deletion. We did not find substantial differences in results, and we report multiple imputed data unless specifically indicated.

In Figure 1 the HAQ, PCS, MCS, and EQ-5D lines, and in Figure 2 the HAQ and PCS lines, were based on predictions from the mixed model, using the fixed-portion linear predictor plus contributions based on predicted random effects. The predictions were smoothed using kernel-weighted local polynomial regression. The distribution curves at the y-axis of Figure 2 represent the distribution at treatment time 0, and were calculated by kernel density estimates.

For ease of interpretation of Table 3 data, estimated predicted values of HAQ, PCS, MCS and EQ-5D are presented at time points of -10 and +10 years, based on adjusted regression analyses.

#### Validation dataset

To understand if the annual rate of change in the outcome variables reported in this study was consistent with clinical practice, we obtained a second dataset of Health Assessment Questionnaire (HAQ-II) data from 847 RA patients seen during ordinary care in a five-rheumatologist clinical practice. The HAQ-II is a 10-item questionnaire that was based on the HAQ. Its scores are essentially the same as those of HAQ. The Pearson correlation coefficient between the HAQ and HAQ-II is 0.85, and Lin's concordance coefficient is 0.85 [33]. The difference between HAQ and HAQ-II mean is 0.01 units.

We also used this data set to develop an algorithm for estimating DAS28 scores from linear regression analysis of DAS28 on HAQ-II, pain, and patient global. Because the validity of standard errors was not an issue, we used all 3,450 observations from the 847 RA patients. The R-square of the model was 0.39. The algorithm was: Estimated DAS28 = 1.877544 + (HAQ-II * 0.1382091) + (VAS pain * 0.0849938) + (VAS patient global * 0.2230887). Substituting the HAQ for the HAQ-II, we calculated an estimated DAS28 in the NDB dataset. This is a rough estimate, and should only be used for putting the NDB data into perspective.

All data were analyzed using Stata 11.0 (Stata Corporation, College Station, TX, USA). Statistical significance was set at the 0.05 level.

The study was carried out in compliance with the Helsinki Declaration, and was approved by the Institutional Review Board of the St. Francis Regional Medical Center, Wichita, KS, USA. All patients signed an informed consent.

## Results

At entry into the NDB, the median duration of RA for study participants was 9.7 years and the mean HAQ (1.1), PCS (35.8), and EQ-5D (0.73) scores were abnormal, particularly in comparison with age- and sex-matched persons in the general population for PCS and EQ-5D, as shown in Table 4. MCS scores, however, were similar in RA and the general population. In addition, the standard deviations and inter-quartile ranges of these four study measures were large, indicating heterogeneity of health status in RA. Methotrexate (MTX) and biologics were used by 60.8% and 44.5% of participants over the duration of the study. The lifetime exposure to disease modifying anti-rheumatic drugs (DMARDs) was 92.3%, and to triple therapy (methotrexate + sulfasalazine + hydroxychloroquine) was 3.7%.

**Table 4:** Characteristics of 18,485 patients with rheumatoid arthritis at study entry unless otherwise specified

*Variable*	*Patient Data*			*Community Norms*	
			
	Mean (SD)	IQR	Range	Mean (SD)	Range
HAQ	1.06 (0.73)	1.00 (0.50 to 1.62)	0.00 to 3.00		
PCS	35.8 (11.0)	34.7 (27.3 to 44.1)	6.5 to 69.4	46.2 (3.4)	40.2 to 55.3
MCS	49.0 (11.3)	51.4 (40.7 to 58.0)	7.6 to 75.2	50.8 (1.46)	45.0 to 52.7
EQ-5D	0.73 (0.19)	0.78 (0.69 to 0.83)	-0.11 to 1.00	0.83 (0.03)	0.79 to 0.93
					
Age (years)	59.9 (13.0)				
Sex (% male)	23.3				
Non-Hispanic White (%)	89.8				
High school graduate (%)	89.4				
College graduate (%)	26.3				
Income (median $US)	35,000				
RA duration (median IQR) years	9.7 (4.4 to 18.1)				
Study duration (years)	3.7 (3.2)		0.5 to 11.0		
Study duration biologic comparison) (years)	6.1 (3.0)		1.0 to 11.0		
Comorbid conditions (none) (%)	27.2				
Comorbid conditions (1) (%)	27.1				
Comorbid conditions (≥ 2) (%)	45.8				
Satisfied or very satisfied with health (%)	51.5				
MTX (%)	47.8				
MTX (anytime in study) (%)	60.8				
Prednisone (%)	40.5				
Biologic (anytime in study) (%)	44.5				
DMARD use (lifetime) (%)	92.3				
Triple therapy (lifetime) (%)	3.7				

HAQ, Health Assessment Questionnaire; PCS, SF-36 Physical Component Summary; MCS, SF-36 Mental Component Summary; RA, rheumatoid arthritis; MTX, methotrexate; DMARD, Disease-Modifying Anti-Rheumatic Drug; triple therapy, methotrexate (MTX) + hydroxychloroquine (HCQ) + sulfasalazine (SSZ).

Community norms are age and sex-adjusted to the NDB study population.

### How does health status change over time?

Figure 1 displays the average course of health status in RA patients over a period of 60 years (solid line). The long-dashed line represents expected values based on normative age and sex adjusted data from the general US population, except for HAQ where normative data are not available.

The four scales demonstrate that, *on average*, profound loss of health status occurs immediately at the onset of RA compared with expected values in the population. For the three scales with physical predominance (PCS, EQ-5D, HAQ), there is a generally linear loss of health status over the next 60 years. However, the rate of loss is only slightly increased compared with the loss of health status that is associated with aging in the general population. There is no increase in the rate of loss of health status over time compared with the general population as measured by the MCS. Indeed the MCS in RA is indistinguishable from population normative data. No population normative data are available for the HAQ, but its pattern of loss is similar to that of PCS and EQ-5D. These data can be summed up as follows: on average, loss of health status occurs immediately, at the onset of RA, and most of the further deterioration in health status that occurs is the deterioration expected by aging independent of RA duration. MCS is an exception to the general observation of loss of health status over time. *Mental health *generally improves slightly over time, and hardly differs from population values.

While the three PCS, EQ-5D, and HAQ graphs show substantial loss of health status over 60 years, the actual mean annual loss is very small, virtually imperceptible. Table 1 and Figure 1 provide information about the rate of progression of RA over the lifetime of the illness. From its intercept, the HAQ increases by 0.016 units per year, the PCS by 0.125 units, and the EQ-5D by 0.001 units per year. It is important to recognize that Figure 1 speaks to the average course of RA. However, the standard deviations and interquartile range (IQR) for all of the measures in Figure 1 are quite wide. For example, as shown in Table 4, the interquartile range (IQR) of the HAQ is 0.50 to 1.625 and the IQR of the PCS is 27.3 to 44.1. This large dispersion is also shown well in the distribution curves at time zero for the HAQ and PCS in Figure 2.

### What is the contemporary rate of change in health status in RA?

We examined the rate of change in health status in detail during the 11-year period of NDB observations (1998 to 2009), using the time metric of *time in study *(11 years), and adjusting for age, comorbidity, year of RA onset, sex, education, and household income (Table 2). During this time, very slight changes in health status were observed. HAQ scores worsened, and PCS, MCS, and EQ-5D scores improved. The annual increase (worsening) in HAQ score was 0.013 (95% CI 0.010, 0.015) units, which is equivalent to a 10-year change of 0.13 units. The annualized improvement rates were PCS 0.035 (95% CI 0.001, 0.069), MCS 0.039 (95% CI 0.008, 0.069), and EQ-5D 0.001 (95% CI 0.001, 0.002). These improvement rates are so small that they can be considered to represent a stable or no-change condition.

When only patients never treated with biologics were considered (Table 2) HAQ scores worsened by 0.016 per year (95% CI 0.013, 0.019), but no significant changes were seen for PCS, MCS, or EQ-5D. Finally, we examined patients who had received biologics during their period of observation in the NDB. The HAQ worsened by 0.010 units per year (95% CI 0.007, 0.013), PCS improved by 0.068 (95% CI 0.022, 0.114) units per year, MCS improved by 0.044 (95% CI -0.003, 0.090), and EQ-5D improved by 0.002 (95% CI 0.012, 0.003) units per year. These data also suggest that patients who received biologics had a course that was more favorable than those not treated with these agents.

### What is the effect of biologic therapy and its cost?

To assess the effect of biologic therapy, we determined annual rates of progression up to the time of receipt of biologic therapy and the rates following administration of such therapy in patients who switched to a biologic while being followed in the NDB. These data are shown graphically in Figure 2 and in detail in Table 3. The mean duration of time on biologics from time 0 was 3.6 years, (median (IQR) 2.8 (1.0 to 5.8) years) during a mean follow-up from time 0 of 4.3 (3.8 (1.5 to 6.5)) years). The DAS28 estimated score immediately prior to biologic start was 3.2, with 41.2% having estimated scores <3.2 and 18.9% have estimated DAS28 scores ≥5.1.

At study closure, 78.8% of patients who started biologics were still receiving them. The annualized rate differences, or benefit for biologic treatment compared to non-biologic treatment, were for HAQ, -0.029 (95% CI -0.023, -0.034), for PCS, -0.532 (95% CI -0.634, -0.430), for MCS, -0.096 (95% CI -0.197, 0.005), and for EQ-5D, -0.005 (95% CI -0.008, -0.002). As is suggested by the rates, the scores at times -10, 0, and +10 years for the combined treatment group differed only slightly (Table 3). In addition, the distribution plots at the y-axis of Figure 2 show that there was wide variability in scores in patients who received biologics. Overall, the effect of biological therapy, while easily discernible, was slight.

We further examined the course of patients who started biologics and remained on (on) them as well as patients who discontinued biologics (DC), as shown in Table 3. As might be expected, patients who started and remained on biologic treatment throughout the study had a more favorable course. For example, they had a mean HAQ score reduction of 0.033 units compared with a decrease of 0.020 units for those discontinuing therapy.

We determined the direct treatment costs and direct total medical costs for each year prior to and after treatment start. Table 3 presents the annualized costs adjusted to 2007 US dollars; and these costs are summed in Figure 2 and estimated for the 10 years before and the 10 years after treatment start. The annualized treatment costs for all patients post-biologic start was $20,401 (95% CI 20,226, 20,576) and the total cost was $24,369 (95% CI 21,172, 24,565). Compared with pretreatment years, annualized treatment costs were $15,720 (95% CI 15,390, 16,051) and total costs were $15,914 (95% CI 15,543, 16,285) greater in biologic treated patients.

### Are the results similar to the progression rates in the clinic: external validation of HAQ progression?

To understand whether the rates of progression were similar in the NDB surveys and the clinic, we calculated the annual rate of HAQ-II increase from 847 RA patients with 6,444 observations during ordinary care in a five-rheumatologist clinical practice (Arthritis and Rheumatology Clinics of Kansas (ARCK)). Each patient had at least one year of follow-up and three clinic visits. The median (IQR) duration of RA at study start was 10.1 (2.9 to 13.0) years. At the last observation, the mean (standard deviation (SD)) age of patients was 60.6 (13.4) years, the HAQ-II was 1.0 (SD), pain was 4.4 (2.6), the DAS28 score was 3.15 (SD), and 57.3% of patients were satisfied or very satisfied with their health. The mean ESR was 20.9 mm/hr, and there were 2.0 swollen and 2.0 tender joint, using the 28 joint count method of the DAS28. Fifty-nine percent used biologics during the study period. Only the HAQ-II was available because the PCS, MCS, and EQ-5D are not ordinarily obtained in clinical practice. The annualized rate of HAQ-II progression was 0.018 (95% CI 0.001, 0.036) units per year, a rate similar to the overall progression rate of 0.013 obtained in the current study (Table 2), though slightly higher, perhaps reflecting the difference between survey and clinic patients.

## Discussion

There are several major findings from this study with respect to RA in general. First, the onset of RA causes, on average, an immediate and substantial reduction in health status, as measured by HAQ, PCS, and EQ-5D scores (Figure 1). Second, after onset, the progression of dysfunction in treated *RA *is very slow. Third, the further decrement in health status over time is only slightly greater than that in the general population, differing primarily by the initial differences between RA and those without RA. Fourth, the variance in any health status measure, at any moment in time, is large as shown in Table 4 and Figure 2.

But perhaps the most important finding of this study was the limited effect of biologic therapy on measures of health status and function. We did find that biologic therapy altered the rate of decrement in health status as measured by the differences in the pretreatment and post-treatment rates of progression. When this effect is expressed in terms of a more manageable 10-year effect rather than a 1-year effect, it can be seen that the estimated 10-year improvement following biologic therapy was 0.29 units for the HAQ, 5.3 units for the PCS, and 0.05 units for the EQ-5D. These values slightly exceed the levels considered to represent minimal clinically important change for these variables [34,35], but are almost six times less than the biologic treatment effect on HAQ identified in a recent meta-analysis comparing methotrexate with biologics in RA of less than three years duration [36]. We also found that the estimated annual increase in total medical and total treatment costs for the second 10-year period compared with the first was close to $16,000.

By contrast, randomized clinical trials of biologics almost universally find changes that we identified over 10 years as occurring over the course of the 6- or 12-month clinical trial [1]. When clinical trial results are extrapolated in cost-effectiveness analyses, it is presumed that the 6- to 12-month improvement will continue for most patients, and that patients who fail to improve will discontinue therapy [37,38]. The degree of improvement found in clinical trials as well as the presumption of continued improvement lead to cost per QALY estimates between $40,000 to $68,000 for biologic therapies [15,37,39,40]. Although we did not do formal cost-effectiveness analyses, our data suggest cost-per-QALY (or incremental cost-effectiveness ratio) may be 5 to 10 times more than calculated in models based on trials.

Patients in the community differed in other ways from patients in clinical trials. Not only did our patients not do as well as those in clinical trials, they also started therapy with much better health status, a mean HAQ score of 1.1 (Table 3) and an estimated DAS28 score of 3.2, and they generally did not discontinue biologics for lack of meeting HAQ improvement criteria (HAQ improvement ≥0.25). Using the DAS28 estimation algorithm derived from ARCK data, we estimate that 41.2% of our patients starting biologics already had DAS28 scores of <3.2 at the time they started a biologic; 18.9% were estimated to have DAS28 scores ≥5.1 at that time point, an indicator of high disease activity.

We believe that the results of our study can be understood as representing the incremental benefit of biologic therapy when it is added to close to optimum non-biologic therapy, therapy that can include DMARDs, NSAIDs, and systemic and intra-articular corticosteroids. Patients in our study were treated by rheumatologists and had received DMARD therapy prior to starting biologics. So we may presume that they received contemporary, specialist, and perhaps close-to-optimum therapy.

A recent study from Moreland et al. sheds light on the incremental benefit in efficacious combination therapies and strategies [41]. They compared the response of 755 methotrexate (MTX) naïve patients with very active RA (mean DAS28 = 5.8) and an RA duration of less than or equal to three years who were treated in a randomized clinical trial with immediate vs. step-up strategy with MTX, etanercept and triple DMARD in four arms: immediate MTX + etanercept or triple therapy; step-up from MTX to MTX+ etanercept or to triple therapy. Patients on combination therapy improved most by six months of treatment, but after two years, there was no significant difference between the groups in outcome measures, and the DAS28 at study closure was 3.0 [41]. Data such as these suggest that improvement can be expected when biologics are added to patients being appropriately treated with DMARDS, but that such improvement is limited.

The Moreland et al. study is particularly relevant because its add-on etanercept treatment arm is similar to the add-on therapy received in our study as part of ordinary clinical care. Based on a conversion algorithm, we estimated that patients in our study had DAS28 scores of approximately 3.2 at biologic start. In addition, in the 721 patients in the ARCK validation dataset the measured DAS-28 score was 3.3 (SD 1.3). Thus the mean DAS28 scores in treated RA may range, approximately, from values around 3.0 in the aggressively treated Moreland et al. study, to 3.3 in the clinical patients in the community, and to 3.2 estimated in the NDB surveys. These observations add support to the idea that there is a general, average level of disease activity that occurs in the presence of contemporary RA treatment.

In a similarly relevant study, Ma et al. performed a meta-analysis of treatments in patients with less than three years of RA (15 studies, 4,200 patients), comparing MTX monotherapy with combination DMARDs and with combined DMARD-biologic therapy [36]. At entry, the DAS score was *high *(4.3 to 6.2). HAQ improvement in the combined DMARD group was -0.17 (95% CI -0.33, -0.01) units and vs. the DMARD/biologic group was -0.16 (-0.26, -0.04) units compared with methotrexate monotherapy. As with the Moreland et al. study [41], they found no benefit with combination therapy compared with methotrexate plus biologics.

An additional example of additive treatment response comes from the SWEFOT (Swedish Pharmacotherapy) trial where 487 patients with RA of less than one year were treated with methotrexate for three to four months [42]. A favorable response (DAS28 ≤3.2) was noted in 145. Of 258 without a favorable response, 130 were allotted randomly to triple therapy DMARD therapy and 128 to infliximab. Twenty-five percent of patients allocated sulfasalazine and hydroxychloroquine achieved the primary outcome compared with 39% assigned infliximab. At entry the HAQ score was 1.3, but final scores were not reported. Thus, these data also suggest that biologic therapy conveys a small incremental benefit to monotherapy and combination DMARD therapy.

It is of interest that although triple therapy has been found to be effective in the above studies, only 3.7% of patients in our study had ever used triple therapy. In addition, the median duration of RA at the inception of our study was 9.7 years, so we could not specifically address the recommendation of the American College of Rheumatology (ACR) that biologics combined with MTX should be the initial therapy for active early RA [43]. However, our results, small incremental benefit from biologics, are more in line with the recommendations of NICE (National Institute for Health and Clinical Excellence) in the United Kingdom (UK) that suggest treating early RA first with DMARD therapy [44].

The NICE report also addresses mild or less active RA, indicating that 'all trials of DMARDs have had active disease as an inclusion criterion ... Studies [are] needed to determine whether it would be safe/effective for people with mild disease to be observed over time without DMARD therapy, or with monotherapy, unless their disease becomes more aggressive. It may be that combination therapies are not appropriate for all people with mild RA.'

Biologic therapy in the US is approved for use in moderate or severe RA, often interpreted as use in those with treatment failure. But the definition of treatment failure is flexible, and dependent on cost, availability, expectations, and satisfaction with health status, in addition to physician and laboratory measurements. Following NICE severity guidelines, the 7,083 biologic-treated patients in the British Society of Rheumatology Biologic Registry (BSRBR) had very active RA at biologic start [45]. Their mean HAQ score was 2.1 compared with 1.1 in the NDB, the UK EQ-5D 0.30 vs. 0.73 (EQ-5D US), and the DAS28 6.7 vs. 3.2 (estimated). For the patients described in Table 4, 9.3% had HAQ scores ≥2.0, 9.2% had EQ-5D UK scores ≤0.3, and <1% had an estimated DAS28 score ≥6.7. Thus the patients in our current study, who are generally representative of RA in the community in the US including those who received biologic therapy, are systematically different from patients in the BSRBR and, generally, from participants in randomized clinical trials. While we applaud the elegant studies of Brennan et al. [45] and others in patients with active RA, it is not clear that their results can be extrapolated to the population of biologic users in the US, including those who appear to continue biologic therapy despite the absence of moderate or good European League Against Rheumatism (EULAR) response [45].

One of the other main findings of this study was the nature of and the rate of progression of loss of health status. Regardless of whether we measured health status with the HAQ, PCS or EQ-5D, we found that health status was lost very early in the course of RA. This is not surprising because pain is the greatest contributor to global health status. What may be surprising, however, is that on average 84% of the HAQ score at 10 years and >95% of the PCS score change occurs immediately around the onset of RA (Table 1). These findings comport with the slow rate of loss of health status that we noted. We found the yearly HAQ progression rate to be 0.016 (0.015, 0.017) over the duration of RA, with an intercept of 0.84. The one-year rate in the ARCK clinical group was 0.018 (0.001, 0.036), and in the patients in Table 3 was 0.032 (0.27, 0.36) in biologic treated patients prior to biologic start and 0.003 (0.000, 0.006) following the start of therapy. It seems possible that the slow rate of progression we noted after RA onset may be due to increasingly effective therapy with methotrexate and other biologic and non-biologic DMARDs.

Our study approached RA and treatment outcomes in a manner different from that of clinical trials. The main outcome of clinical trials is usually comparative improvement, while the main interest in longitudinal outcome studies is the level of health status. In addition, instead of DAS scores or ACR improvement criteria [12] in patients with active RA in clinical trials, we examined the effect of RA and its treatment on health status in *average *patients in the US community. While we were unable to measure RA activity, we measured the consequence of RA activity and RA treatment with the HAQ and SF-36, widely accepted, validated tools that effectively predict important RA outcomes, such as mortality, work disability, household income, and other patient outcomes [46-49]. These measures incorporate RA activity, in particular RA pain, with cumulative RA effect and damage. Bansback et al. indicate that that the HAQ is the 'primary clinical measure for use in economic evaluations as it is measured in almost all clinical studies, and is closely correlated to health utilities, mortality and costs.' [14]

It is often not recognized that low levels of disease activity (DAS28 scores of approximately 3.0 to 3.2) do not mean no activity. But, as the ARCK data show, at a DAS28 score of 3.15, the average RA patient had a HAQ-II score of 1.0, VAS pain score of 4.4, and two swollen and two tender joints; 42.7% patients were not satisfied with their health. In the current NDB study, we found profound impairment in PCS, HAQ, and EQ-5D despite a low estimated DAS28 score.

There are a number of advantages to the methods of our study. Because patients and their physicians decide when to start and stop therapy without regard to trial needs, external validity is high. Indeed, the finding that the average patient starting therapy was much less severe than in clinical trials speaks to increased generalizability. One advantage of the method used in our study is that it measures long-term treatment effects, and is not sensitive to flares and flare/response changes. As observational studies can be sensitive to regression to the mean [50], change in slopes within patients provides protection against that bias.

There are a number of limitations to this study. We relied on self-report of start and stop dates of treatment, and these dates might be reported inaccurately to us. To account for this potential problem we performed sensitivity analyses under a series of assumptions, including calculations using a pre-treatment start time six months earlier. We did not observe differences in results. Even so, it is possible that some attenuation of effect of biologic therapy might have occurred because of our methods.

Another point that should be considered is that implicit in the rate change data in the biologic treatment group are two assumptions that cannot be tested; first, that the increased rate of progression noted in the pre-treatment group would have continued had they not been treated; second that the treatment received resulted in the change in progression rate. While these assumptions seem reasonable, they represent counterfactuals that cannot be tested.

Patients in our study had more education, and higher levels of household income than seen in the general population. In addition, minorities were under-represented in this study. These are characteristics of survey participants [51]. Because we had measures of these socio-demographic covariates, we adjusted for them in our analyses, and it is unlikely these study characteristics altered the results of our study. Finally, we were concerned that data from clinic patients and survey patients might be different because of the difference in setting. However, we were assured by noting that the rate for progression of HAQ disability was similar in the ARCK clinic as in the surveys.

A final limitation of this study is that it represents only US data. There are no strict standards for biologic use in the US. Patients in this study with mild RA received biologics, a use that would be considered inappropriate in many other countries, given the cost of these therapies.

The data of this study raise a number of other questions about health status in RA and its measurement. RA results in progressive damage to joints, a fact that is verified by radiographic damage and joint replacements that may become necessary later in the course of the illness. From observations such as these, one may come to the reasonable conclusion that there is a progressive loss function and health status. The problem with this interpretation is not that it is necessarily wrong, but that it doesn't, on average, comport with the results of validated health status measures that show that the profound loss of health status occurs very early in RA. Our data suggest that there might be a substantial disconnect between damage and health status, and that pain and difficulty and uncertainty, the burden of RA, may impact measured self-reported health status more than damage.

## Conclusions

RA onset caused an immediate and substantial reduction in physical but not mental health status. Thereafter, the progression of dysfunction in RA was very slow (HAQ 0.016 units and PCS -0.125 units annually, only slightly worse than the age and sex-adjusted US population. We estimated biologic treatment to improve HAQ by 0.29 units, PCS by 5.3 units, and EQ-5D by 0.05 units over a 10-year period. The estimated incremental 10-year total direct medical cost for this benefit was $159,140. Biologic therapy retards RA progression, but its effect is far less in the community than is seen in clinical trials. Consequently, cost-effectiveness is substantially less than that estimated from clinical trial data. The study results represent the incremental benefit of adding biologic therapy to optimum non-biologic therapy, and are in accord with recent add-on studies.

## Abbreviations

ACR: American College of Rheumatology; ARCK: Arthritis and Rheumatology Clinics of Kansas; BSRBR: British Society of Rheumatology Biologic Registry; DAS28: disease activity score-28; DC: discontinued biologics; DMARDs: disease modifying anti-rheumatic drugs; EQ-5D: EQ-5D health utility scale; EULAR: European League Against Rheumatism; FDA: Food and Drug Administration; HAQ: Health Assessment Questionnaire; HAQ-II: Health Assessment Questionnaire-II; ICE: multiple imputation by chained equations; IQR: interquartile range; MCS: Mental Component Summary score; MTX: Methotrexate; NDB: National Data Bank for Rheumatic Diseases; NICE: National Institute for Health and Clinical Excellence; PCS: Physical Component Summary Score; QALYs: Quality Adjusted Life-Years; RA: rheumatoid arthritis; SD: standard deviation; SF-36: Medical Outcome Study Short Form 36; SWEFOT: Swedish pharmacotherapy; Triple therapy, methotrexate + sulfasalazine + hydroxychloroquine; UK: United Kingdom; US: United States; VAS: visual analog scale.

## Competing interests

The NDB has received past or current funding from Abbott, Amgen Bristol-Meyers Squibb, Centocor, Pfizer, and UCB. The current study was the idea of the authors, and received no funding from past or present funding sources. The authors collected, analyzed, and wrote the study entirely by themselves. Kaleb Michaud received partial funding from the Arthritis Foundation's New Investigator Award and NIH ARRA grant #1RC1AR058601-01.

## Authors' contributions

FW drafted the manuscript, and participated in the conception, design, data collection, and data analysis of the study. KM helped in the drafting of the manuscript, and participated in the conception, design, data collection, and data analysis of the study. All authors read and approved the final version of the manuscript.
